# Computation-aided studies related to the induction of specialized metabolite biosynthesis in microbial co-cultures: An introductory overview

**DOI:** 10.1016/j.csbj.2023.08.011

**Published:** 2023-08-16

**Authors:** Tomasz Boruta

**Affiliations:** Lodz University of Technology, Faculty of Process and Environmental Engineering, Department of Bioprocess Engineering, ul. Wólczańska 213, 93-005 Łódź, Poland

**Keywords:** Co-culture, Specialized metabolites, Data analysis, Molecular networks, Genome mining, Optimization

## Abstract

Co-cultivation is an effective method of inducing the production of specialized metabolites (SMs) in microbial strains. By mimicking the ecological interactions that take place in natural environment, this approach enables to trigger the biosynthesis of molecules which are not formed under monoculture conditions. Importantly, microbial co-cultivation may lead to the discovery of novel chemical entities of pharmaceutical interest. The experimental efforts aimed at the induction of SMs are greatly facilitated by computational techniques. The aim of this overview is to highlight the relevance of computational methods for the investigation of SM induction via microbial co-cultivation. The concepts related to the induction of SMs in microbial co-cultures are briefly introduced by addressing four areas associated with the SM induction workflows, namely the detection of SMs formed exclusively under co-culture conditions, the annotation of induced SMs, the identification of SM producer strains, and the optimization of fermentation conditions. The computational infrastructure associated with these areas, including the tools of multivariate data analysis, molecular networking, genome mining and mathematical optimization, is discussed in relation to the experimental results described in recent literature. The perspective on the future developments in the field, mainly in relation to the microbiome-related research, is also provided.

## Graphical Abstract

**Figure:**
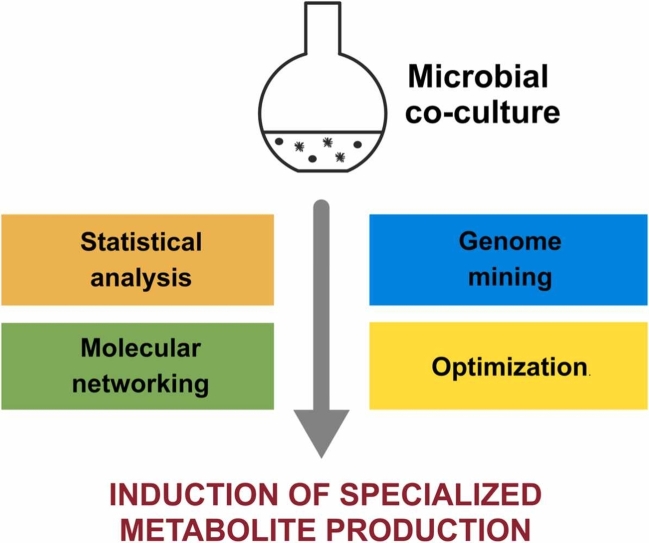

## 1 Introduction

The drug discovery initiatives are based on two fundamental approaches to find novel lead compounds. The first strategy relies on the generation of structural diversity through high-throughput methods of chemical synthesis, whereas the alternative option relies on the exploration of biosynthetic landscapes hidden in natural sources, including bacteria, fungi and plants [1], [2], [3]. The demand for new drugs remains unabated, being fueled by the emerging microbial and viral threats, as exemplified by the Covid-19 pandemic [4], [5] and the antibiotic-resistant bacterial strains [6], [7]. It is thus not surprising that even now, almost a century after Fleming’s discovery of penicillin [8], microbial metabolism continues to be perceived as a promising reservoir of bioactive molecules. For instance, the widely investigated bacterial genus *Streptomyces*, mostly recognized for its remarkable capability to biosynthesize various antibiotics, remains an exciting research subject for chemists and microbiologists [9]. Even though drawing the clear boundary between primary and specialized metabolism (also referred to as secondary metabolism in the previous literature reports) may in some cases be problematic, and the roles of specialized metabolites (hereafter referred to as SMs), are often a matter of speculation, the widely accepted view is that these molecules are not directly involved in cellular growth and energy generation. Instead, they provide the producing strain with certain advantages over its competitors in an ecological niche [10], [11], [12]. The bioactivity exhibited by SMs can be tested in a medical context and, in the case of promising results, given further consideration during the drug development projects [13]. Importantly, the fact that a given microbial strain is genetically equipped to biosynthesize a particular SM does not guarantee that the metabolite will be produced under laboratory conditions. The activation of biosynthetic gene clusters (BGCs), i.e*.*, genomic regions that encode the machinery responsible for SM biosynthesis, requires specific environmental signals associated with nutrient availability, cellular stress, elicitors, growth conditions, and other factors [14], [15], [16]. Microbial co-cultivation is a popular approach applied for the activation of BGCs and “awakening” cryptic biosynthetic pathways. Co-cultures are inexpensive and do not require the involvement of genetic engineering. This unconventional method of laboratory cultivation aims to mimic the conditions experienced by microbial strains while interacting with other organisms in natural environments. The idea behind the co-cultivation approach is to influence SM production capabilities via the physical and/or chemical interactions between microorganisms [17], [18]. There are several possible outcomes of co-cultures as far the biosynthesis of SMs is concerned: (a) the production of a given SM may be observable both in mono- and co-cultures at comparable levels, (b) the metabolite may be present both in mono- and co-cultures albeit at significantly different levels (i.e*.*, up- or down-regulated), (c) the production may be recorded exclusively in monocultures, or (d) the SM is induced (*de novo* induced or “awaken”) due to co-cultivation and observed solely under the conditions of co-culture [19]. Additionally, if the induced SMs have not been previously described, they are regarded as newly discovered chemical entities and can be further characterized. Importantly, the relevance of microbial co-cultures is observed far beyond the subject of SM discovery. This strategy is also employed for the investigation of microbial communities [20], [21] and the development of synthetic microbial consortia [22], [23]. Progressing from studying the axenic cultures (i.e*.*, the monocultures) towards exploring the complexity of co-cultures can be perceived as a “paradigm shift” in microbiological sciences [24]. It is important to note that microbial co-cultures are associated with several bioprocess-related issues [25], [26], [27], [28], [29], [30]. The difference in growth rates may lead to the scenario in which the faster-growing species outcompetes its co-culture partner and, as a result, the slower-growing microbe is eventually eliminated. Several methods were tested to address this problem. For example, the initial inoculation ratio of two species can be optimized by considering the relative growth rates and product titers [25]. It is also possible to fine-tune the co-culture dynamics by adjusting abiotic factors, e.g., medium osmolality or agitation conditions [26]. Designing the co-culture initiation method is particularly challenging in the case of filamentous microorganisms, since their co-cultivation can be started with the use of spore suspensions or precultures and these two inoculation approaches may lead to markedly different outcomes in terms of SM production [27]. Furthermore, the production of SMs in co-cultures is greatly dependent on time. While for some SMs the titers increase continuously over the course of co-cultivation, it is possible that a given SM is detectable over a relatively short time interval, as was already demonstrated in several studies related to microbial co-cultures [26], [28], [29].

Since the development of modern experimental tools relies heavily on mathematics and computer science, it is not surprising that the advancements in the field of co-cultivation-based SM discovery are also catalyzed by the application of computational methodologies. Building biological knowledge based on the omics datasets requires rigorous and high throughput approaches [31] and this fact is greatly exemplified by the co-culture-related research. As discussed in the present review, computational tools are employed not only in the interpretation of omics data but they also complement the efforts of metabolite annotation, the optimization of fermentation conditions and, finally, the identification and characterization of BGCs in producer strains.

This review briefly introduces the fundamental aspects of SM induction in microbial co-cultures and gives an overview of computation-aided research studies in the field. The goal is to provide conceptual primer and a “bird’s eye view” on the subject without focusing on the underlying mathematical intricacies. The text also highlights the relevance of computational methods for the investigation of SM induction in co-cultures.

## 2 Statistics-based detection of specialized metabolites induced in co-cultures

The fundamental task of SM discovery is to use the available experimental tools to activate the production of novel microbial SMs, identify the newly discovered chemical entities and determine their bioactivities and potential use. In this context, the reason for applying the co-culture approach is to induce (or “awake”) the production of the otherwise non-produced molecules. When the necessary condition of SM production is met and the levels of metabolites are above the detection limits, the next task is to differentiate between the metabolic repertoires displayed in mono- and co-cultures. The common approach used to investigate the metabolic fingerprints of the samples is to apply mass spectrometry (MS), which is typically coupled with liquid (LC-MS) or gas (GC-MS) chromatography to provide an upstream separation of molecules prior to their MS analysis [19]. In principle, for the well-defined peaks and relatively strong signals it may be possible to detect the *de novo* induction of SMs manually, i.e*.*, use the recorded mass chromatograms to notice the fact that a given metabolite is biosynthesized exclusively under co-culture conditions. However, in most cases it is not feasible due to the relatively small amounts of metabolites in the sample, peak overlapping, data noise and, most importantly, a large number of analyzed datasets originating from numerous experimental replicates. To address these difficulties and extract biologically relevant information from large datasets, a rigorous procedure of data processing and analysis is required. In the field of metabolomics, i.e*.*, the large-scale study of the metabolic composition of the cell [32], the key findings regarding the SM induction are expected to be supported by thorough statistical analysis and reported in a transparent way with the aid of graphical visualization [33]. The importance of computational framework associated with the data analysis step of metabolomics experiment cannot be overstated [34]. Chemometric tools [35], typically embedded within the metabolomics software [36], are used to extract the meaningful information from the multidimensional datasets involving the metabolite *m/z* values, signal intensities, peak areas, retention times, fragmentation patterns, etc., for numerous experimental variants and replicates. To detect the “mono- vs. co-culture” differences in terms of SM production within the sets of metabolomics data, the statistical tools of multivariate data analysis are applied [37], such as principal component analysis (PCA) [38], partial least squares coupled to a discriminant analysis (PLS-DA) [39], [40], and orthogonal projections to latent structures coupled to a discriminant analysis (OPLS-DA) [41]. Generally, the mathematical transformation leading to dimensionality reduction of data is performed and the score plots are used to illustrate the groupings and outliers. In other words, the data is transformed into a lower-dimensional space in a way that aims to preserve the information represented by the original dataset and the differences between the samples are then assessed. Finally, the SMs responsible for these differences are detected, possibly revealing the SMs which are produced in co-culture but not in the corresponding monocultures [42]. It is important to note that the pattern-recognition methods of multivariate data analysis can be divided into two categories, namely the unsupervised and supervised methods, as explained in the previous review of Alonso et al. [43]. If the unsupervised approach is followed, the direct identification of similarity patterns is performed in the original data without considering the types or classes of study samples. This type of analysis is often performed as a first exploratory step in metabolomics studies [19]. Among the unsupervised methods, PCA is a commonly used chemometric approach capable of providing an overview of complex experimental data [44]. In PCA, the metabolic features are transformed into a set of linearly uncorrelated variables called principal components. This transformation is carried out in a way that maximizes the variance represented by the first component, while the subsequent components are associated with an increasingly reduced variance [43]. By contrast, the supervised methods, e.g., PLS-DA or OPLS-DA, involve the use of sample labels to identify the metabolic patterns associated with the variables of interest, while down-weighing the variance associated with the features that are not associated with these variables [43]. As pointed out by Gromski et al. [39], the PLS-DA approach is suitable for the analysis of noisy and highly collinear data, which is often recorded over the course of metabolomics studies. In addition, it offers the statistic measures (e.g., loading weight or regression coefficient) that can be employed to identify the key phenotypic variables. In the OPLS-DA method, developed as an extension to the PLS-DA approach, an integrated orthogonal signal correction filter (OSC) allows for separating the predictive from non-predictive variation [41]. Finally, it should be mentioned that rigorous validation is always required to avoid statistically unreliable conclusions when analyzing metabolomics data, therefore, employing multiple data analysis methods in a single study is not an uncommon practice [45].

For clarity, the overview of studies that employed the multivariate statistics to analyze the production of metabolites in microbial co-cultures [46], [47], [48], [49], [50], [51], [52], [53], [54], [55], [56], [57], [58], [59], [60], [61], [62], [63], [64], [65], [66], [67], [68], [69] is listed in Table 1.

**Table 1:** Overview of studies on the production of metabolites in microbial co-cultures involving multivariate analysis of metabolomics data.

**Co-cultivated microorganisms**	**Type of co-culture**	**Employed methods of multivariate analysis**	**Selected highlights of the study**	**Reference**
Plant-derived, clinical or soil fungal isolates, (mainly *Fusarium* sp.) co-cultured in various combinations	fungus vs. fungus	PCA, PLS-DA	Development and validation of high-throughput metabolomics methodology for the analysis of fungal co-cultures; Screening of more than 600 co-cultures. Demonstration that many fungi produce SMs in response to co-cultivation	[46]
*Micromonosporaceae* vs. *Mycobacterium* sp. strain WMMA-183 or *Rhodococcus* sp. strain WMMA-185	bacterium vs. bacterium	PCA	Induction of SMs in co-cultures involving 12 strains of *Micromonosporaceae*	[47]
*Trichophyton rubrum* vs. *Fusarium solani*	fungus vs. fungus	POCHEMON	Development and assessment of POCHEMON methodology	[48]
*Pseudomonas aeruginosa* ATCC 27853 vs. *Aspergillus fumigatus* AZN 8196 (a clinical isolate)	bacterium vs. fungus	PLS-DA	Demonstration that the volatile organic compound (VOC) profiles in the co-cultures of *P. aeruginosa* and *A. fumigatus* are markedly different from the ones exhibited in the corresponding monocultures	[49]
*Pseudomonas aeruginosa* ATCC 27853 vs. *Aspergillus fumigatus* AZN 8196 (a clinical isolate); *Aspergillus clavatus* Sin141, (soil isolate) vs. *Fusarium* sp. PS54743	bacterium vs. fungus; fungus vs. fungus	ANOVA-POCHEMON	Development and assessment of ANOVA-POCHEMON methodology	[50]
*Eutypa lata* vs. *Botryosphaeria obtusa*	fungus vs. fungus	AMOPLS	Induction of several volatile and non-volatile metabolites; Demonstration of antifungal activity of 2-nonanone	[51]
21 marine-adapted fungal isolates vs. *Pseudomonas syringae* or *Ralstonia solanacearum* or *Magnaporthe oryzae* or *Botrytis cinerea*	bacterium vs. fungus; fungus vs. fungus	PCA, PLS-DA	Annotation of molecular clusters of metabolites induced via co-cultivation	[52]
*Ganoderma applanatum* CGMCC No. 5.249 vs. *Trametes versicolor* CGMCC No. 12241	fungus vs. fungus	PCA	Identification of a novel phenyl polyketide up-regulated in co-cultures	[53]
110 fungal pairs involving 16 wood-decaying basidiomycetes (including *Trametes robiniophila* Murr and *Pleurotus ostreatus*)	fungus vs. fungus	PCA	Induction of a family of novel sesterterpenes (postrediene A, B and C) produced by *P. ostreatus* in co-cultures	[54]
*Streptomyces* sp. strain AV05 vs. *Fusarium verticillioides* strain 1163	bacterium vs. fungus	PCA, PLS-DA	Examination of the influence of co-cultivation on the endometabolome of *F. verticillioides*; Recorded overproduction of several metabolites in co-cultures	[55]
*Streptomyces lunalinharesii* A54A vs. *Rhizoctonia solani* CMAA 1589	bacterium vs. fungus	PCA	Induction of SM production in *S. lunalinharesii* via the co-cultivation approach; Inhibition of *R. solani* growth in co-cultures with *S. lunalinharesii*	[56]
*Streptomyces* sp. WU20 vs. *Aspergillus sclerotiorum* DX9 vs.	bacterium vs. fungus	PCA, OPLS-DA	Enhancement of notoamides biosynthesis in *A. scletotiorum* due to the production of cyclo(Pro-Trp) by *Streptomyces* sp.	[57]
*Bacillus subtilis* vs. *Aspergillus sydowii*	bacterium vs. fungus	PLS-DA	Induction of 25 metabolites via co-cultivation, including 4 novel molecules	[58]
*Fibrobacter succinogenes* strain UWB7 vs. *Anaeromyces robustus* or *Caecomyces churrovis*	bacterium vs. fungus	PCA	Enrichment of fungal metabolites in co-culture; Indication that anaerobic gut fungi may produce antimicrobials in co-cultures with rumen bacteria	[59]
*Cophinforma mamani* E224 vs*. Fusarium solani* PLR2	fungus vs. fungus	PLS-DA	Induction of 5 metabolites via co-cultivation	[29]
*Bacillus subtilis* BR4 vs. *Pseudomonas aeruginosa* ATCC 27853	bacterium vs. bacterium	PCA	Demonstration that *B. subtilis* BR4 inhibits quorum-sensing in *P. aeruginosa* ATCC 27853	[60]
*Lacticaseibacillus casei* Zhang vs. *Lactiplantibacillus plantarum* P8	bacterium vs. bacterium	PCA, PLS-DA	Induction of 4 metabolites, including mangiferin, via the co-cultivation approach	[61]
*Bacillus subtilis* CGMCC 13141 vs. *Aspergillus sydowii* CPCC 401353	bacterium vs. fungus	PLS-DA	Induction of 15 SMs in optimized co-cultures	[62]
*Aspergillus oryzae* vs. *Zygosaccharomyces rouxii*	fungus vs. fungus	PCA, OPLS-DA	Demonstrated the metabolic differences between the co-cultures and the corresponding monocultures of *A. oryzae* and *Z. rouxii* (grown on solid medium)	[63]
*Aspergillus oryzae* vs. *Zygosaccharomyces rouxii*	fungus vs. fungus	PCA, OPLS-DA	Demonstrated the metabolic differences between the co-cultures and the corresponding monocultures of *A. oryzae* and *Z. rouxii* (grown in liquid medium)	[64]
*Chlorella pyrenoidosa* vs. *Ganoderma lucidum*	alga vs. fungus	PLS-DA, OPLS-DA	Enhancement of triterpenoids biosynthesis via co-cultivation	[65]
*Prorocentrum lima* vs. *Aspergillus pseudoglaucus*	dinoflagellate vs. fungus	PCA, OPLS-DA, POCHEMON	Demonstration of the co-culture-related up-regulation of the dinoflagellate toxins okadaic acid and dinophysistoxin 1	[66]
*Bacillus* sp. strain LPPC170 vs. *Fusarium kalimantanense* strain LPPC130	bacterium vs. fungus	PLS-DA	Identification of volatile organic compaounds (VOCs) responsible for the inhibition of growth of *F. kalimantanense*	[67]
*Alternaria alternata* or *Alternaria tenuissima* vs. *Trichoderma harzianum* JF309 or *Trichoderma koningii* GIM3.137 or *Trichoderma harzianum* GIM3.442 or *Trichoderma harzianum* Q710613 or *Trichoderma atroviride* Q710251 or *Trichoderma asperellum* Q710682 or *Trichoderma virens* Q710925	fungus vs. fungus	PCA, OPLS-DA	Demostration that *Trichoderma* spp. affect the metabolome of *Alternaria* and that *T. atroviride* Q710251 is capable of biotransforming alternariol into its less toxic hydroxylated form	[68]
*Bacillus licheniformis* GZ241 or *Bacilllus subtilis* GZ237 or *Bacillus amyloliquefaciens* GZ121 vs. *Eurotium amstelodami* GZ23	bacterium vs. fungus	PCA, PLS-DA	Co-culture-related enhancement of the production of several SMs, including nummularine B, lucidenic acid E2, elatoside G, aspergillic acid, copaene, and pipecolic acid	[69]

PCA, principal component analysis; PLS-DA, partial least squares coupled to a discriminant analysis; OPLS-DA, orthogonal projections to latent structures coupled to a discriminant analysis; AMOPLS, ANOVA Multiblock OPLS method; POCHEMON, projected orthogonalized chemical encounter monitoring; ANOVA-POCHEMON, analysis of variance-projected orthogonalized chemical encounter monitoring

Even though the aforementioned methods of multivariate analysis, namely PCA, PLS-DA and OPLS-DA, are the most frequently used statistical tools in the co-culture studies related to the induction of SMs [70], the alternative strategies are also available, including the ANOVA Multiblock OPLS method (AMOPLS) [71]. Apart from revealing the biologically relevant findings, the co-culture-related studies also provide important methodological insights. For instance, Adnani et al. [47] used the co-cultures of *Verrucosispora* sp. (strain WMMB-224) with *Mycobacterium* sp. and *Rhodococcus* sp. to evaluate the influence of data scaling on the results of co-culture analysis by using PCA. In the absence of scaling, the analysis was reported to be dominated by high intensity signals, while the unique yet low-intensity ones went undetected. While the application of unit variance scaling led to the unwanted effect of removing the intensity variable, the Pareto scaling proved to be a well-balanced approach that ultimately allowed to detect a unique SM in one of the investigated co-cultures [47]. In a different study, Swift et al. [59] encountered the case in which the separation between the analyzed datasets was apparent in a three-dimensional but not in the two-dimensional PCA scores plot. Such nuances must be approached individually in each study to avoid the misinterpretation of data.

Notably, the statistical methods mentioned so far are universally employed in metabolomics even outside the topic of microbial co-cultivation. By contrast, the chemometric tool known as projected orthogonalized chemical encounter monitoring (POCHEMON) was developed specifically for the purpose of studying microbial interactions and metabolite production in co-cultures [48]. The co-culture of *Trichophyton rubrum* and *Fusarium solani*, isolated from the infection known as onychomycosis, was used as a model to demonstrate the advantages of the POCHEMON approach over PCA, PLS-DA and ANOVA in terms of representing the microbial interactions in co-cultures while taking into the consideration the systematic variability between the co-cultures replicates [48]. Later, the combined methodology known as ANOVA-POCHEMON was developed by Geurts et al. [50] to address interaction dynamics in co-cultures. Considering the published reports, however, the traditional multivariate analysis methods, such as PCA and PLS-DA, are still predominantly applied in the co-culture/SM-focused studies (Table 1). The popularity of these approaches may be associated with their long history of use and the universal applicability across the spectrum of metabolomics topics. So far, the use of POCHEMON has been reported among the researchers responsible for the development of this methodology and the documented applications of POCHEMON [48], [50], [66] are still rare. Nevertheless, the statistical methods tailored specifically to be applied in co-culture studies, such as POCHEMON, can be expected to be used more and more frequently in the upcoming SM investigations. It is important to mention that POCHEMON can be downloaded and run in the freely available R environment (https://gitlab.univ-nantes.fr/bertrand-s-1/pochermon). Sharing the source code with the scientific community is a prerequisite for the successful use of novel computational solutions.

It should be noted that metabolomics is not the sole approach applied for the discrimination between the metabolic profiles exhibited in mono- and co-cultures. Since it is based on the ionization of molecules prior to their detection, mass spectrometry is effective provided the analyzed metabolites are ionizable [72]. Complementing metabolomics with nuclear magnetic resonance (NMR) was demonstrated by Nguyen et al. [55], who employed PCA and PLS-DA in the analysis of metabolites in the co-cultures of *Fusarium verticillioides* with *Streptomyces* sp. strain AV05. In a different study, the approach of NMR-based metabolomics coupled with multivariate data analysis led to the identification of several metabolites formed by *Aspergillus niger* in response to *Streptomyces coelicolor* [73]. Finally, metabolomics can be employed in concert with transcriptomics to investigate the biosynthetic response of microorganisms to co-cultivation [74].

## 3 Annotation of specialized metabolites with the use of molecular networking

Sorting out the MS data corresponding to the SMs produced exclusively under co-cultivation conditions should ideally be followed by the structural characterization of the detected molecules. This task is far from trivial, as it generates substantial expenses, consumes time and, most importantly, requires analytical expertise and specialized equipment. Prior to entering the laborious efforts of structural elucidation, it is now possible to automatically annotate SMs by comparing the experimental MS results with the library-deposited datasets. As reviewed by de Jonge et al. [33], the computational metabolite annotation tools typically rely on the assignment of similarity scores between the mass fragmentation spectra (MS/MS). The calculation of similarity scores is in turn based on the chosen similarity metrics. The most common scoring approach, referred to as the cosine score, involves the transformation of MS/MS spectra into vectors and subsequently calculating their dot-product. However, there are many alternative scoring metrics available, including the variations of the cosine score and the entropy-based score. Currently, the comparison of MS/MS datasets can be also performed with the use of machine learning approaches. Finally, the MS/MS spectra can be organized into groups to facilitate their annotation [33]. Applying the computational approach is a convenient way of starting the identification of SMs by formulating the data-driven hypotheses regarding the structural characteristics of the investigated molecules. One can notice an analogy between the genome sequencing projects and the exploration of chemical space in microbial cultures. Once the genome sequence is known, the automatic annotation based on bioinformatics is routinely performed to assign the putative functions to identified genes [75], [76], [77], [78], [79]. Using the computational pipelines is easy, fast and inexpensive, as opposed to the laborious manual corrections and community-driven curation that follow the step of algorithmic genome annotation. The philosophy behind the automatic functional annotation is that sequence similarity between the genes suggests their functional similarity. In other words, the higher the similarity scores, the higher the probability that the two compared genes are functionally related [80]. Such comparisons are possible due to the availability of biological databases that can be used as references whenever a newly sequenced genome is analyzed. A similar comparison-based approach can be employed in metabolomics to facilitate the SM discovery workflows. The concept of community-wide sharing of mass spectra and other experimental datasets within the publicly available repositories is exemplified by the development of Global Natural Products Social Molecular Network (GNPS) [81], [82], an open-access knowledge base of MS/MS data. Developing and analyzing the MS libraries within the GNPS opens the door for the fast and automated annotation of SMs based on MS/MS data. Importantly, it can also be employed for the dereplication of known molecules from the analyzed mixtures [83]. Identification of previously discovered chemical entities is performed to avoid investing time and resources on SM rediscovery. Among several available GNPS capabilities, molecular networking (MN) is a popular computational tool frequently used in the studies on SM production in microbial co-cultures. While the functional similarity of genes is usually reflected by the sequence similarity, the idea behind MN is that the structurally similar molecules exhibit similarities in terms of their fragmentation patterns. These patterns are represented by the MS/MS spectra of investigated molecules, which can be algorithmically compared and scored, leading to a network representation of their relatedness. As a result, the families of structurally similar SMs are depicted visually as clusters within the network [84], [85]. MN can be thus perceived as a way of translating the chemical similarity information (reflected by MS/MS patterns) into a network representation, in which mass spectra are represented by nodes and related nodes are connected by edges. Hence, MN provides a structure-related perspective for the annotation of newly discovered SMs prior to performing the time-consuming and costly structural studies. Importantly, the MN approach complements the multivariate data analysis in the detection of co-culture-unique metabolites. For example, in the study involving the use of GNPS platform, Asamizu et al. [86] described the unique clade of metabolites found in the co-cultures of *Streptomyces hygroscopicus* HOK021 and *Tsukamurella pulmonis* TP-B0596 but not in the corresponding monocultures. In a different study, Oppong-Danquah et al. [52] challenged a set of marine-adapted fungal isolates originating from the Windebyer Noorgainst lake with phytopathogenic fungi (*Magnaporthe oryzae*, *Botrytis cinerea*) or bacteria (*Pseudomonas syringae*, *Ralstonia solanacearum*). The GNPS-based MN procedure resulted in the annotation of several molecular clusters (involving polyketides, terpenes, alkaloids and other classes of SMs), some of which were recorded solely for the co-cultures. Importantly, a number of the analyzed chemical entities were regarded as “putatively new metabolites”, as they did not exhibit fragmentation pattern similarities with the deposited database spectra [52]. This topic was further explored in a recent work focused on the co-cultures of *M. oryzae* with a marine fungus representing the *Cosmospora* genus [87]. The MN-involving investigation showed that, compared with the monocultures, the confrontation of fungal strains led to the expansion of chemical space, as reflected by the increased size of several clusters in the assembled molecular network. In addition, the co-culture-related induction of isochromanones was recorded [87]. Sun et al. included the GNPS tools into their computational workflow aimed at the identification of metabolites in “*Aspergillus sydowii* vs. *Bacillus subtilis*” co-cultures and the accuracy of the suggested *in silico* approach was verified by NMR analysis [58], [62]. Notably, as many as 25 metabolites were detected exclusively in co-cultures and 4 of these molecules were marked as novel chemical entities [58]. The utilization of computational workflow for metabolite annotation in co-cultivation experiments was also reported by Maimone et al., who studied the co-cultures of *Streptomyces lunalinharesii* A54A with the phytopathogenic species *Rhizoctonia solani* and employed the GNPS library for the putative identification of desferrioxamines and anisomycin [56]. The examples of studies involving the application of GNPS tools and MN analysis include the co-cultures of *Pleurotus ostreatus* vs. *Trametes robiniophila* Murr [54], *Aspergillus sclerotiorum* DX9 vs. *Streptomyces* sp. WU20 [57], *Aspergillus terreus* C23–3 vs. *Aspergillus unguis* DLEP2008001 [88], *Trametes versicolor* vs. *Ganoderma applanatum* [53], *Cophinforma mamani* E224 vs. *Fusarium solani* PLR2 [29], *Aspergillus* spp. NCA257 vs. *Cladosporium* sp. NCA273 or *Aspergillus* sp. NCA276 [30], and *Eurotium amstelodami* vs. three species of *Bacillus* [69]. It should be mentioned, however, that the application of network-based analysis of mass spectra was demonstrated by the developers of GNPS in the context of SM/co-culture research even before the formal introduction of the GNPS infrastructure. Traxler et al. [89] employed spectra clustering and spectral network assembly for the investigation of interactions of *Streptomyces coelicolor with* other *Actinomycetes* during their co-cultivation on agar medium. Briefly, spectral networking led to the discovery of a family of acylated desferrioxamines, which were produced and secreted by *S. coelicolor* in response to the siderophores generated by the accompanying *Actinomycete*. It was also demonstrated that the repertoire of SMs biosynthesized by *S. coelicolor* was dependent on the neighboring bacterium [89]. All in all, considering the above-referenced examples, it is evident that MN and the GNPS-based computational tools are relevant for the annotation of SMs in microbial co-cultures. Currently, the toolbox of MN methods is under constant development and incorporates more and more functionalities yet to be tested in the context of SM production in co-cultures [90], [91]. As the MN approach relies on automated workflows, publicly available databases and freely accessible web-based services, its popularity among the members of SM/co-culture community can be expected to grow. Finally, apart from the purpose of metabolite annotation, network representation can be used to explore the chemodiversity within a certain set of chemical entities. Instead of using the MS/MS spectra, such networks can be based on the chosen physicochemical properties of the analyzed molecules, e.g., their molecular weight and lipophilicity. This was demonstrated in the review of Arora et al. [92], who analyzed 82 published articles and assembled the structure similarity network based on "Tanimoto structure similarity index" for 259 metabolites reported to be induced via the co-cultivation approach.

## 4 Identification of biosynthetic gene clusters in producer strains

The identification of co-culture-induced SMs is accompanied by the identification of their microbial source. In the case of molecules produced solely in co-cultures, finding the producer among the interacting strains is not straightforward. This task can be accomplished via genetic analysis, i.e*.*, proving that the genetic inventory required for the biosynthesis of the metabolite is present in the genome of the strain suspected of being the producer [42]. In this context, the approach known as genome mining is widely used to detect and annotate the BGCs present in microbial genomes by using computational tools [93], [94], [95]. As far as the studies on SM induction in co-cultures are concerned, the platform known as antiSMASH (antibiotics & Secondary Metabolite Analysis Shell) is routinely chosen as the software tool for putative BGCs identification and analysis [96]. It uses profile hidden Markov models (pHMMs) to identify the biosynthetic functions represented by the BGCs and defines these functions within the sets of “rules”. These manually curated “rules” enable the *in silico* identification of BGCs within the analyzed genomic segments. The current version of antiSMASH encompasses the “rules” for 81 types of BGCs, including the ones encoding non-ribosomal peptide synthetases (NRPSs) and polyketide synthases (PKSs) [96]. An example of antiSMASH application was presented by Asamizu et al. [86] in relation to the co-cultivation of *S. hygroscopicus* HOK021 and *T. pulmonis* TP-B0596. The co-cultivation approach led to the induction of several SMs, including enterobactin, platensimycin, thioplatensimycin, and harundomycin A. Afterwards, the antiSMASH analysis was performed and the BGCs for enterobactin and platensimycin were identified in the genome of *S. hygroscopicus* HOK021. Since no similar BGCs were detected in the genome of *T. pulmonis* TP-B0596, it was concluded that *S. hygroscopicus* HOK021 was the source of enterobactin and platensimycin induced in the co-cultures [86]. In a different study, Shin et al. [97] discovered a novel cyclic peptide, dentigerumycin E, in the co-cultures of *Bacillus* sp. GN1 and *Streptomyces* sp. JB5, the strains which had been previously isolated from an intertidal mudflat. The authors expected *Streptomyces* sp. JB5 to be the producer of *dentigerumycin* E based on previous literature reports. To support this hypothesis, they employed antiSMASH to identify a putative BGC associated with the production of dentigerumycin E in the sequenced genome of *Streptomyces* sp. JB5. As a result, a PKS-NRPS gene cluster was found, which was predicted to generate a cyclic peptide with a sequence consistent with the structure of a newly discovered SM [97]. Recently, interesting results related to BGCs and co-cultures were reported by Ninomiya et al. [98], who employed antiSMASH as a component of their experimental workflow. The BGCs were first identified within the genomes and then the transcriptomic analysis was conducted to determine the clusters that were upregulated in co-cultures relative to the monoculture controls. It was noted that the main product of the *ors* cluster in *Aspergillus nidulans* was different in the “*A. nidulans* vs. *A. fumigatus*” and “*A. nidulans* vs. *Streptomyces rapamycinicus*” co-cultures. In the former case, the production of diphenyl ether was recorded, whereas in the latter variant the biosynthesis of orsellinic acid took place. So, the product of the cluster depended on the accompanying microorganism [98]. Hu et al. [99] investigated the inhibitory effects exerted by *Bacillus cereus* CF4–51 on the plant pathogen *Sclerotinia sclerotiorum*. The co-cultivation experiments revealed that the volatile organic compounds (VOCs) generated by *B. cereus* CF4–51 disturbed the development and damaged the hyphal structures of *S. sclerotiorum*. Fengycin was among the SMs identified in the biosynthetic repertoire of *B. cereus* CF4–51 and this finding was further confirmed via the antiSMASH analysis, which revealed the presence of a BGC related to fengycin biosynthesis in the genome of this bacterium [99]. The use of antiSMASH for the identification of candidate BGCs was also reported by Shen et al. [54] and Kontomina [100] et al. in their broad-range co-cultivation studies encompassing 110 fungal pairs and 5144 bacterial pairs, respectively.

While the use of antiSMASH in the SM/co-culture studies can be seen as widespread, it should be mentioned that the identification of producer strains by using the *in silico* genome mining approach is not free of limitations. The first issue, already mentioned by Knowles et al. [42], is the necessity of genetic tractability of the co-cultivated strains. In other words, one cannot perform the sequence similarity searches unless the genetic sequence of the investigated strains is available. The second limitation stems from the fact that there are still relatively few reference BGCs that have been experimentally characterized and verified in terms of their corresponding SMs. Using the automated annotation pipelines is always associated with a risk of generating incorrect functional assignments by referring to the unconfirmed data that was not yet manually curated. Finally, in the case of newly discovered SMs that have not been yet biosynthetically characterized in any microorganism, only the BGCs corresponding to the structurally similar compounds (if available) can be employed as reference sequences. Alternatively, the computational predictions regarding the structure of SMs associated with a given cluster can be taken into consideration [97]. If the BGC of a novel SM is identified and confirmed, it can be then subjected to further investigations, e.g., the studies on the regulatory mechanisms behind the SM production, the elucidation of the SM biosynthetic pathway or, provided there is any interest in producing greater amounts of the new SM, heterologous expression in one of the workhorse production strains [94].

## 5 Optimization of co-cultivation conditions

If the co-culture yields novel SMs of potential biotechnological interest and the genetic basis for their biosynthesis is deciphered, the methods of heterologous expression and cell factory design may be employed to construct the engineered producer strains capable of providing industry-level yields and titers of target molecules. As soon as the relevant biosynthetic genes are transferred from a natural producer to a new microbial host, the incredibly complex regulatory system governing the SM biosynthesis in the wild-type strain becomes practically irrelevant and the expression can be fine-tuned via genetic manipulations of the engineered microbe. Notably, the methods of microbial co-cultivation aided by the computational tools are becoming increasingly important in the context of metabolic engineering and synthetic biology. Their applicability was demonstrated in the work of Jones et al. [101], who used the co-culture of engineered *E. coli* strains to produce flavonoids. The astonishing 970-fold improvement of product titer was recorded relative to the previously shared monoculture results. The study involved the initial optimization of several factors (e.g., carbon source, inoculation ratio, and temperature) and the subsequent empirical scaled-Gaussian modelling to fit the data and determine the optimum. Since then, the design of artificial microbial consortia [102] has gathered much attention, also in the context of developing dedicated computational frameworks built to facilitate the experimental efforts [103]. While microbial co-cultures may be sometimes perceived as “black boxes” that yield novel molecules, the emerging rational approach of “co-culture engineering” employs the tools of metabolic engineering and synthetic biology to “make use of engineered interdependencies” [23] between the co-cultivated microorganisms. According to Liu et al. [104], co-culture engineering “offers multiple environments for enzyme expression, creates physical barriers that insulate bioprocesses from one another and distributes the burden of heterologous expression between members, imparting advantages to produce complex natural products”. A convenient way to classify microbial communities used in biotechnology is to consider the “nature of their assembly”, as described by Ibrahim et al. [105], who defines “natural consortia” (i.e*.*, isolated from natural environments), “artificial consortia” (i.e*.*, assembled artificially), and “synthetic consortia” (i.e*.*, involving genetically modified strains). In addition, the term “engineered microbial consortium” is used to describe a rationally designed system in which complex biological tasks (e.g., the biosynthesis of metabolic precursors) are distributed among microbial populations [106]. While the topic of engineered microbial consortia is beyond the scope of this review, it is worth mentioning that mathematical optimization has also been applied in the context of SM induction resulting from the interactions of wild-type strains in co-cultures. An example of this type of study was provided by Li et al. [107], who employed response surface methodology (RSM) for the optimization of antifungal SM production in co-cultures of *Trichoderma atroviride* and *Bacillus subtilis*. A statistical model was used to optimize the co-cultivation conditions and medium composition. As a result, the stimulation of the production of plant growth promoting and antifungal metabolites was observed. Notably, the optimized co-cultivation variant yielded 8 new SMs [107]. In a different study, Sun et al. [62] followed the RSM approach to optimize the conditions of “*Aspergillus sydowii* vs. *Bacillus subtilis*” co-culture for the production of antibacterial metabolites. This strategy led to the induction of 15 SMs and the increase of inhibition rate against *Staphylococccus aureus* [62]. These two studies demonstrated the fact that the outcome of microbial co-cultivation depends on bioprocess conditions. In the future studies, the chances of discovering novel SMS may be increased by combining the OSMAC (“one strain-many compounds”) approach, which was tested and proven to be effective in the context of SM induction in monocultures [108], with the methodology of microbial co-cultivation. Using a broad collection of microbiological media for the propagation of co-cultures will represent the multi-stimulus approach to uncovering the yet unexplored parts of specialized metabolic networks.

The bioprocess-related studies on microbial co-cultures may involve the routinely performed data processing steps, e.g., function approximation and differentiation for the determination of substrate consumption and metabolite production rates, as was done for the bioreactor co-cultures of *Aspergillus terreus* with *Streptomyces rimosus* [109] and *A. terreus* with *Streptomyces noursei* [110]. In the case of filamentous microorganisms, the quantitative investigation of morphological development is often employed for the monitoring of microbial growth over the course of co-cultivation [111], [112], [113], [114], [115]. For this purpose, the values of morphological parameters (e.g., projected area, elongation, or roughness) are determined with the aid of microscopy and dedicated software tools [116], [117]. So far, the digital image analysis of filamentous morphologies has been performed for the co-cultures propagated in shake flasks [118], [119] and 5-liter stirred tank bioreactors [120], [121].

## 6 Concluding remarks and outlook

The traditional approach to SM discovery involves chemical procedures of extraction, isolation and purification of novel molecules. This “find and grind” [96] workflow can be complemented by the use of computational techniques and unconventional microbiological methods, such as co-cultivation. The present overview focused on four areas related to SM induction in co-cultures that were aided by the application of computational methods, namely the detection of newly induced SMs, their annotation and identification, the search for the BGCs in a producer strain, and the optimization of co-cultivation conditions. It should be mentioned that almost all the computational tools employed in the studies referenced here were not tailored specifically for co-culture-related applications and can be used both for the mono- and co-cultivation experiments. This applies to data analysis methods and molecular networking in metabolomics, the mining of BGCs in genome sequences and mathematical optimization methods. Expanding the toolbox of methodologies capable of addressing the nuances of co-culture experiments (with POCHEMON [48] and ANOVA-POCHEMON [50] serving as examples) and making the development of bioinformatics solutions available for the community would accelerate the discoveries in the field and open the door for comprehensive and broad-scope investigations. Complementing the SM discovery with the in-depth examination of interspecies communication and ecological relations can be expected to take place in concert with the development of novel workflows for the in vivo and *in silico* studies.

The future developments in the field can also be expected to be associated with the analysis of microbiomes and the investigation of SM production in multi-species microbial consortia. The rational design of co-cultivation studies will be fueled by discoveries related to the community-level microbial interactions and the ecology-oriented analyses. This trend can already be observed in recent literature reports. In an important study of Chevrette et al. [122], the three-species bacterial model involving *Pseudomonas koreensis*, *Bacillus cereus*, and *Flavobacterium johnsoniae* was employed to demonstrate that the dynamics of specialized metabolism “depend on community species composition and interspecies interactions”. On a side note, the study involved the use of multivariate data analysis, molecular networking and BGC mining methods, what proves their relevance for the current and future developments in the SM-related investigations.

It may be argued that practically all computational methods employed in the areas of microbiology, biochemistry and molecular biology are to some extent relevant for the SM/co-culture studies, as they facilitate the efforts leading to the improvement of biological knowledge and deeper understanding of molecular mechanisms within the cell. For instance, the computational pipelines widely used in genome sequencing and annotation projects [79] are essential in the process of providing high-quality sequence data required for the genome-scale analysis of BGCs and the monitoring of transcriptional responses to co-cultivation. So, it would be justified to state that the importance of computational methods for the investigation of co-culture-based SM induction reaches far beyond the applications mentioned here.

## Funding

The project was funded by the National Science Centre of the Republic of Poland, grant number 2017/27/B/NZ9/00534.

## Author statement

The submitted work has not been published previously. The submitted work is not under consideration for publication elsewhere. The publication is approved by all authors and the responsible authorities where the work was carried out. If accepted, the submitted work will not be published elsewhere in the same form, in English or in any other language, including electronically without the written consent of the copyright-holder. The Author declares no conflict of interest. The Author declares that generative artificial intelligence (AI) and AI-assisted technologies were not used in the writing process.

## Declaration of Competing Interest

The authors declare that they have no known competing financial interests or personal relationships that could have appeared to influence the work reported in this paper.
